# 3D-bioprinted HepaRG cultures as a model for testing long term aflatoxin B1 toxicity *in vitro*

**DOI:** 10.1016/j.toxrep.2020.11.003

**Published:** 2020-11-21

**Authors:** Konrad Schmidt, Johanna Berg, Viola Roehrs, Jens Kurreck, Munir A. Al-Zeer

**Affiliations:** Department of Applied Biochemistry, Institute of Biotechnology, 4/3-2, Technische Universität Berlin, Gustav-Meyer-Allee 25, 13355 Berlin, Germany

**Keywords:** 3D-bioprinting, Aflatoxin B1, Alternative *in vitro* model, HepaRG liver cells

## Abstract

In recent years 3D-bioprinting technology has been developed as an alternative to animal testing. It possesses a great potential for *in vitro* testing as it aims to mimic human organs and physiology. In the present study, an alginate-gelatin-Matrigel based hydrogel was used to prepare 3D-bioprinted HepaRG cultures using a pneumatic extrusion printer. These 3D models were tested for viability and metabolic functions. Using 3D-bioprinted HepaRG cultures, we tested the toxicity of aflatoxin B1 (10 or 20 μM) *in vitro* and compared the results with 2D HepaRG cultures. There was a dose-dependent toxicity effect on cell viability, reduction of metabolic activity and albumin production. We found that 3D-bioprinted HepaRG cultures are more resistant to aflatoxin B1 treatment than 2D cultures. Although the metabolic activities were reduced upon treatment with aflatoxin B1, the 3D models were still viable and survived longer, up to 3 weeks, than the 2D culture, as visualized by fluorescence microscopy. Furthermore, albumin production recovered slightly in 3D models after one and two weeks of treatment. Taken together, we consider using 3D-bioprinting technology to generate 3D tissue models as an alternative way to study toxicity *in vitro* and this could also provide a suitable alternative for chronic hepatotoxicity studies *in vitro*.

## Highlights

• 3D-bioprinting method to produce 3D HepaRG constructs.
• 3D constructs are more resistant to aflatoxin B1.
• Long-term toxicity assessment is possible.
• An alternative method for animal testing relevance.

## Graphical abstract

**Figure:**
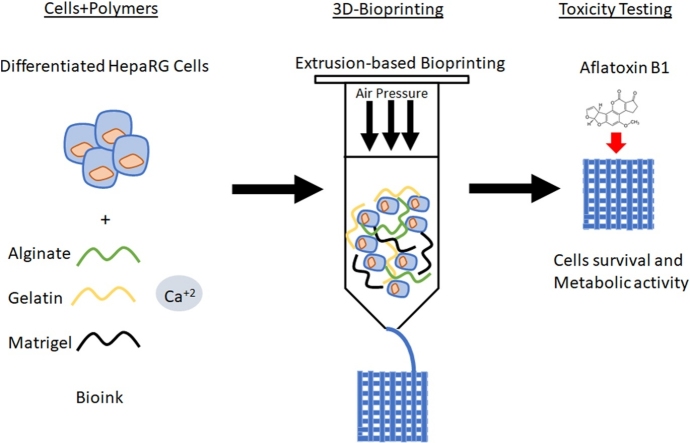

## 1 Introduction

*In vitro* cell cultures and animal experiment models are crucial instruments in basic research and preclinical studies [[1], [2], [3]]. Even though cell cultures and animal models are widely used in the field of toxicology testing, recent advances in 3D-bioprinting technology are emerging as a useful tool for testing of complex cell environments, toxicity and for drug discovery *in vitro* [4,5,42] . Traditional 2D cell culture systems using cell lines or primary cells do not recapitulate the natural three-dimensional microenvironment of the respective human tissue or organ, which in turn leads to changes in gene and protein expression [1,6]. On the other hand, beyond the inherent complexity of animal models, the microenvironments of animal tissues do not efficiently recapitulate many of their human counterparts [7]. The creation of microenvironments *in vitro* that capture some features of human tissues could be enabled by advances in the understanding of 3D-bioprinting [8]. For this reason, 3D-bioprinting is considered a promising tool to study infection, cancer, drug screening, and toxicity testing [9,10]. This can reduce or replace the need for animal testing that does not precisely reflect human responses in terms of toxicology and pathophysiology.

Although 3D-bioprinting is a cutting edge technology used to fabricate 3D models *in vitro* that hold tremendous promise, especially in the fields of biology and medicine, 3D-bioprinting technology and biomaterials are still being fine-tuned [11]. Bioprinting offers great precision that enables the exact spatial and temporal deposition of biological materials, including cells and extracellular matrix in a 3D hierarchal organization to generate artificial 3D models of native tissues. The precise deposition and spatial arrangement of these components can recapitulate the natural architecture and the microenvironments of the tissue *in vitro* [11]. Both natural and synthetic polymers, such as collagen [12], gelatin [13], and alginate [14] *etc.*, are used extensively to fabricate scaffolds for 3D model printing due to on their biocompatibility, printability and biodegradability properties [15].

Several 3D mini-organs and tissue constructs have been printed and fabricated *in vitro,* including skin, heart, bone, cartilage, lung, neurons, and pancreas using different scaffolds and utilizing various 3D-bioprinting approaches [11]. Liver 3D mini-models have also been generated *in vitro* using various tissue engineering techniques including 3D-bioprinting [16]. Various cell models have been used to fabricate 3D liver model *in vitro* including the use of human iPSC-derived hepatocytes [4], HepG2 cells [17], and HepaRG cell line [18]. The HepaRG cell line has been shown to be stable *in vitro*, allowing long-term culture [18]. Liver models using 3D-bioprinted cells are becoming more common in clinical and basic research, including infection, drug discovery, and toxicity testing [19,20]. As the liver plays a central role in metabolism and detoxification of chemicals, 3D-bioprinting possesses great potential to achieve these goals and model complexity that mimics the liver functions *in vitro* [43]. Therefore, 3D-bioprining has the potential to become an important tool for *in vitro* toxicity testing especially for studies of chronic hepatotoxicity against toxins such as aflatoxins. Aflatoxins are secondary fungal metabolites, known as mycotoxins, which are mainly produced by the genus *Aspergillus* [21,22]. They are classified as Group 1 carcinogen by the International Agency for Research in Cancer [23]. Aflatoxins are hepatotoxic and have been implicated in increasing the risk of hepatocellular carcinoma. Aflatoxin B1 has been shown to induce cytotoxicity in HepaRG cells [44]. This hepatotoxicity is mediated by its toxic epoxide, which is produced by CYP1A2 and CYP3A4 [24]. Further, aflatoxin B1 induces loss of cell viability due to the induction of apoptosis in HepaRG cells. This phenotype has been correlated with DNA damage response mediated by p53 signaling [24,25].

In this study, we aimed at optimizing a 3D liver cell model using bioprinting technology with an alginate/gelatin/Matrigel-based scaffold, which can be used for extrusion-based bioprinting of HepaRG cell models for toxicity testing. The 3D liver constructs were generated using a 3D micro-extrusion bioprinter. Viability, cytotoxicity and metabolic activity of the printed constructs were evaluated. Upon treatment with AFB1, cells grown in 2D did not survive the damage induced by the toxin, unlike the 3D-bioprinted constructs. Although AFB1 reduced metabolic activity in the 3D-bioprinted constructs, cells survived the AFB1 toxicity and were still viable, as visualized by fluorescence microscopy. Therefore, a 3D-bioprinted model may pave the way to study the long-term effect of AFB1 and carcinogenesis *in vitro*.

## 2 Materials and methods

### 2.1 Cell culture

HepaRG cells were obtained from Biopredic International (Saint Gregoiré, France). The cells were cultured in William’s E medium (Gibco, Dreieich, Germany) supplemented with 2 mM L-Glutamine (Biowest, Nuaillé, France), 10 % fetal bovine serum (FBS; c.c.pro, Oberdorla, Germany), 50 μM hydrocortisone hemisuccinate (Sigma, Steinheim, Germany), 5 μg/mL recombinant human insulin (PAN Biotech, Aidenbach, Germany), and 1 % penicillin/streptomycin (P/S; Biowest). The cells were cultured at 37 °C and 5 % CO_2_in a humidified incubator for 14 days before differentiation and the medium was renewed every three days. After 14 days, hepatic differentiation was induced by adding 1.7 % DMSO (Sigma) to the culture medium for additional 14 days as previously described [20].

### 2.2 Preparation of cell-scaffold hydrogels

Sodium alginate (4.5 % w/v) and gelatin (6.5 % w/v) powders (Sigma) were dissolved in William’s E medium on a magnetic stirrer at 1250 min^−1^ (overnight at 37 °C). The hybrid alginate -gelatin hydrogel (450 μl) was mixed with liquid Matrigel (200 μl) (Corning, Tewksbury, MA, USA), differentiated HepaRG cells, 1.22 M CaSO_4_(25 μl) (Roth, Karlsruhe, Germany) and William’s E medium containing supplements (325 μl) to obtain the final cell-scaffold mixture bioink composed of alginate (2 % w/v), gelatin (3 % w/v), Matrigel (20 % w/v), 30 mM CaSO_4_ and 7 * 10^6^HepaRG cells/ml, as previously described [26]. Following the initial cross-linking of alginate using CaSO_4_ (8 min after mixing), the cell-scaffold hydrogel was loaded into the printing cartridge (Cellink, Gothenburg, Sweden).

### 2.3 3D bioprinting

The Cellink INKREDIBLE+ 3D-printer was utilized for the bioprinting process which was carried out at room temperature. A rectangular-shaped construct (1 mm height x10 mm width x10 mm length) was designed with regularly spaced pores in a grid-like pattern using Slic3r software. The shape was selected to allow the diffusion of nutrients, oxygen, and metabolites through the constructs. The hydrogel was extruded through a 22 G needle at 10–40 kPa to generate 3D-liver constructs designed by the computer-aided design (CAD) software Rhinoceros5 (Robert McNeel & Associates, Seattle, WA, USA). The printed constructs were further cross-linked using 100 mM CaCl_2_ (Roth) to increase the gelation of alginate. Then, constructs were cultured with William’s E medium supplemented with 1.7 % DMSO, as well as 20 mM CaCl_2_, and incubated at 37 °C and 5 % CO_2_ in a humidified incubator.

### 2.4 Staining with fluorescent DNA dye and immunofluorescence

Printed 3D constructs were fixed for 30 min using 4 % formaldehyde. The constructs were then permeabilized with 1 % Triton-X-100 (Roth) for 1 h at room temperature and the nuclei were stained with Hoechst dye (1 μg/ml) (H33342, AppliChem, Darmstadt, Germany). Cellular distribution was analyzed with the Zeiss Observer. Z1 microscope (Zeiss, Jena, Germany).

### 2.5 The live/dead viability assay

To determine cell viability, we used a commercial Live/Dead assay (Viability/Cytotoxicity kit; ThermoFisher Scientific, Waltham, MA, USA). Briefly, the 3D liver constructs were incubated with 2 μM calcein-AM and 4 mM ethidium homodimer-1 diluted in 1x HBSS (ThermoFisher Scientific) for 15 min (37 °C, 5 % CO_2_). Subsequently, samples were analyzed by fluorescence microscopy (Zeiss Observer. Z1 microscope; Zeiss, Germany).

### 2.6 XTT assay

Metabolic activity of HepaRG cells printed constructs or 2D cultures was determined using the tetrazolium hydroxide salt (XTT) assay according to the manufacturer’s instructions (AppliChem, Germany) at indicated time points. The absorbance at 450 nm, with a reference of 620 nm, was carried out using TriStar Multimode Reader LB942 (Berthold Technologies, Bad Wildbad, Germany). Cell-laden constructs incubated in 10 % Triton-X-100 (Roth), which was diluted culture medium were used as lysis control. Values were normalized to the lysis controls.

### 2.7 Lactate dehydrogenase release

The supernatants from different constructs were collected at each time point, as indicated, and lactate dehydrogenase colorimetric assays (Roche, Switzerland) were carried out according to the manufacturer's instructions. The absorbance at 490 nm, with a reference of 620 nm, was measured with the Sunrise microplate reader (Tecan, Männedorf, Switzerland).

### 2.8 Albumin measurement

The supernatants from 3D constructs or 2D monolayers were analyzed and quantified for albumin secretion using the human albumin enzyme-linked immunosorbent assay (ELISA) kit (Bethyl Laboratories, Montgomery, TX, USA), according to the manufacturer’s instructions. The absorbance at 450 nm, with a reference of 620 nm, was carried out using TriStar Multimode Reader LB942.

### 2.9 Treatment with toxins

Differentiated HepaRG cells were treated with 10, or 20 μM aflatoxin B1 (AFB1) (Sigma-Aldrich). The AFB1 was dissolved in dimethylsulfoxide (DMSO). Control cells were treated with the same volume of DMSO for 1, 2, 5 and 7 days of incubation. For long-term culture cells were treated with single dose 10, or 20 μM AFB1 for one week then cells were cultured for additional two weeks without the toxin. A single dose of doxorubicin (Dox) (Sigma-Aldrich) was used to treat the constructs for 1, 2, and 3 days. Dox was also dissolved in DMSO to a final concentration of 10, or 20 μM.

### 2.10 Statistical analysis

Data were analyzed using Student's *t*-test (GraphPad Prism 6, GraphPad Software, Inc., La Jolla, CA, USA). Data are represented as mean ± SD, p-values are considered significant by *p ≤ 0.05; **p ≤ 0.01; ***p ≤ 0.001; **** p ≤ 0.0001.

## 3 Results

### 3.1 Preparation and characterization of HepaRG cell-scaffold bioink

Bioink optimization is a critical step in bioprinting. To assess the suitability of the hydrogel composed of alginate-gelatin-Matrigel (2 %, 3 %, and 20 % respectively) for 3D-bioprinting, differentiated HepaRG cells were used to generate 3D constructs for toxicity testing studies *in vitro*. The preparation of the printing process was performed as previously described [26]. Briefly, the hydrogel was pre-heated to 37 °C and loaded into a three ml-syringe, cells, Matrigel and CaSO_4_ were loaded into another syringe. Using a Luer-lock adapter, both syringes were connected, and the hydrogels mixed thoroughly. They were then loaded onto a printing cartridge and printed using the INKREDIBLE + bioprinter (Cellink). The optimized hydrogel consisting of alginate-gelatin-Matrigel enabled the printing procedure, and protected the integrity of the 3D constructs, cell viability, and metabolic activity of the cells.

**Fig. 1:**
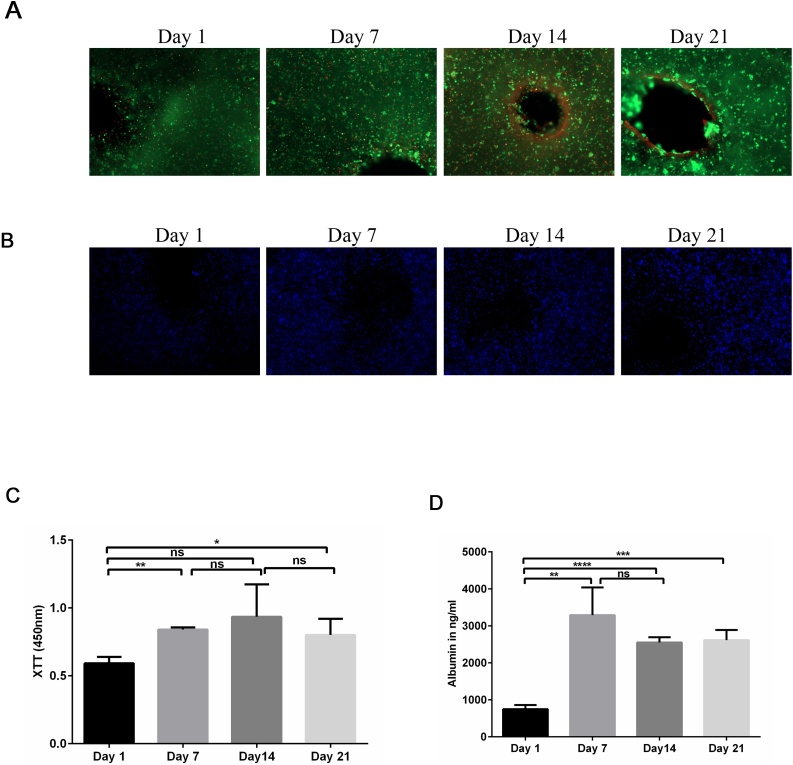
Characterization of bioprinted 3D constructs. (**A**) Qualitative viability staining of the 3D printed mature HepaRG cells in the constructs at the indicating time points using the Live/Dead staining; calcein-AM (live cells in green) and ethidium homodimer-1 (dead cells in red). (**B**) Spatial distribution of mature HepaRG cells in 3D constructs using alginate/gelatin/Matrigel as a bioink at the following time points: 1, 7, 14, and 21 days post-printing visualized by nuclear Hoechst staining (blue). Graphs are representative images of three independent experiments. (**C**) Metabolic activity of mature HepaRG cells in the 3D constructs was determined by tetrazolium hydroxide salt (XTT) assays at the indicated time points post-printing. Absorbance was carried out at 450 nm. (**D**) Albumin secretion of mature HepaRG 3D constructs quantified using enzyme-linked immunosorbent assay (ELISA) analysis at the indicated time points. Results are shown as mean ± SD of three independent experiments. * p ≤ 0.05; ** p ≤ 0.01; *** p ≤ 0.001; **** p ≤ 0.0001.

The viability of the printed cells was confirmed by live/dead staining (Fig. 1A). The 3D constructs were qualitatively analyzed using calcein-AM (green) to stain living cells and ethidium homodimer-1 (red) to stain dead cells after 1, 7, 14, and 21 days of culture, followed by fluorescence microscopy. No obvious increase in the number of dead, ethidium homodimer-1-positive cells, was detected at any of the time points. Furthermore, the number of living, calcein-AM-positive cells, was relatively stable during the entire time course (Fig. 1A). Further, we analyzed the spatial distribution of the differentiated HepaRG cells within the 3D constructs using fluorescence microscopy. First, the nuclei were stained with Hoechst DNA dye (blue) and the distribution of the cells was visualized at the indicated time points (Fig. 1B). Cells were homogeneously distributed in the printed 3D constructs without significant differences during the time course of the experiment (Data not shown). Taken together, we conclude that the 3D-printed liver constructs are viable, and the cells are distributed evenly throughout the hydrogel.

Using XTT assays, we quantified the metabolic activity of the HepaRG cells in the 3D printed constructs (Fig. 1C). Consistent with the cell viability obtained using the live/dead assay, the metabolic activity in the 3D constructs increased significantly at day 7 and 21 compared to day 1 (Fig. 1C). Furthermore, we analyzed albumin secretion using ELISAs to determine the metabolic activity of the hepatic cells in 3D constructs (Fig. 1D). Albumin secretion is one of the main characteristics of hepatocytes and its production reflects a specific-function of liver cells. The amount of albumin secreted from 3D printed cells increased over time, and this increase was statistically significant at days 7, 14 and 21 (Fig. 1D). Taken together, our results showed substantial increases in cell viability and metabolic activity over time in the 3D cell culture system.

### 3.2 Assessment of aflatoxin B1 toxicity using HepaRG 2D culture

**Fig. 2:**
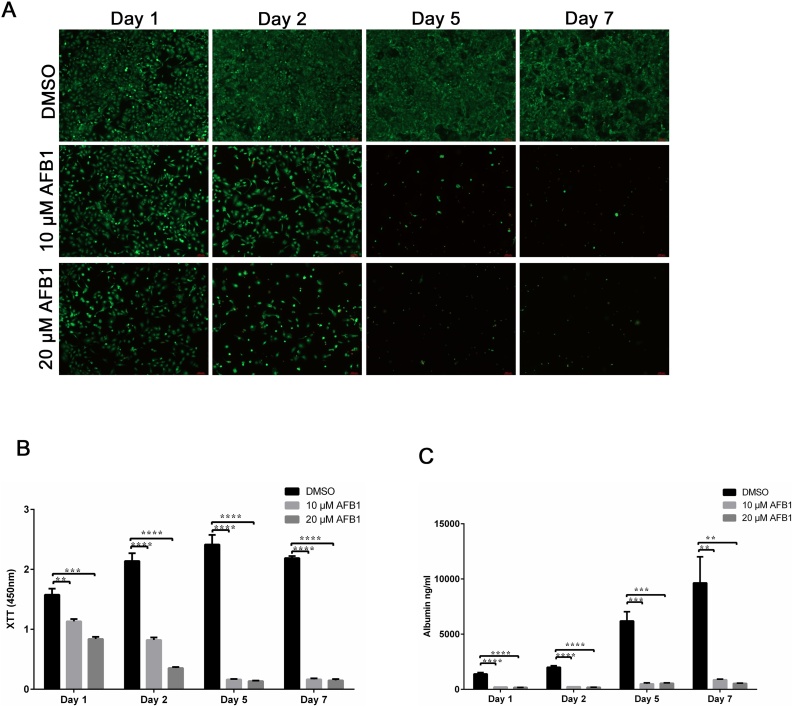
Assessment of aflatoxin B1 toxicity on liver cells grown as 2D monolayers. (**A**) Monolayers of mature HepaRG cells, treated with DMSO, 10 μM AFB1, and 20 μM AFB1 at the indicated time points were tested for cell survival using live-dead staining. Live cells fluoresce green, whereas dead cells fluoresce red. Graphs are representative images of three independent experiments. (**B**) Metabolic activity of mature HepaRG cells grown in 2D upon AFB1 treatment at the indicated time points. Activities were determined using tetrazolium hydroxide salt (XTT) assays. Absorbance was carried out at 450 nm. (**C**) Albumin levels of mature HepaRG cells in 2D monolayers upon AFB1 exposure were quantified at the indicated time points using ELISA analysis. Results are shown as mean ± SD of three independent experiments. * p ≤ 0.05; ** p ≤ 0.01; *** p ≤ 0.001; **** p ≤ 0.0001.

To study the sensitivity of cells cultured in 2D to toxicity stemming from AFB1, HepaRG cells were seeded onto a12-well plate at a density of 5*10^5^ cells per well. Cells were then treated with a single dose of AFB1 (10 and 20 μM) or DMSO, which served as a control. Following treatment with AFB1, cells were stained and analyzed for cell viability using live/dead stain and fluorescence microscopy. AFB1 treatment (10 μM) resulted in a reduction of viable cells (calcein-AM positive-cells shown in green) after 24 and 48 h (Fig. 2A). Similar results were obtained from the cells treated with 20 μM AFB1; however, the toxic effect of AFB1 was more pronounced after 48 h post-treatment (Fig. 2A). After five and seven days of treatment, cell cultures showed signs of severe cytotoxicity, as the number of viable cells detected among the AFB1-treated cells was very low or undetectable, in contrast to the control DMSO-treated cells that reached confluency within two days after initial seeding (Fig. 2A).

Next, we quantified the reduction of the tetrazolium salt XTT by dehydrogenase enzymes to determine the metabolic activity of the 2D HepaRG cell cultures upon AFB1 treatment (Fig. 2B). Consistent with the cell viability obtained using the Live/Dead stain, AFB1 treatment resulted in a significant reduction of the metabolic activity of the 2D cultures after 24 and 48 h (Fig. 2B). Further, the metabolic activity in the 2D cell cultures treated with AFB1 decreased over time and was statistically no longer measurable on days five and seven of culture.

To assess the impact of AFB1 on the hepatic metabolism of HepaRG cell culture, albumin secretion was analyzed using ELISAs. In the DMSO-treated differentiated HepaRG cells, the amount of secreted albumin increased over time; however, the increase was only statistically significant at day five (Fig. 2C). In contrast, AFB1 treatment inhibited albumin production strongly, without obvious differences in albumin secretion between the different concentrations tested (Fig. 2C). Taken together, AFB1 toxicity showed substantial increases in toxicity over time in the 2D cell culture system. This toxicity was characterized by reduced metabolic activity and reduction of albumin secretion over the time course of the experiment.

### 3.3 Assessment of aflatoxin B1 toxicity using 3D printed HepaRG constructs

**Fig. 3:**
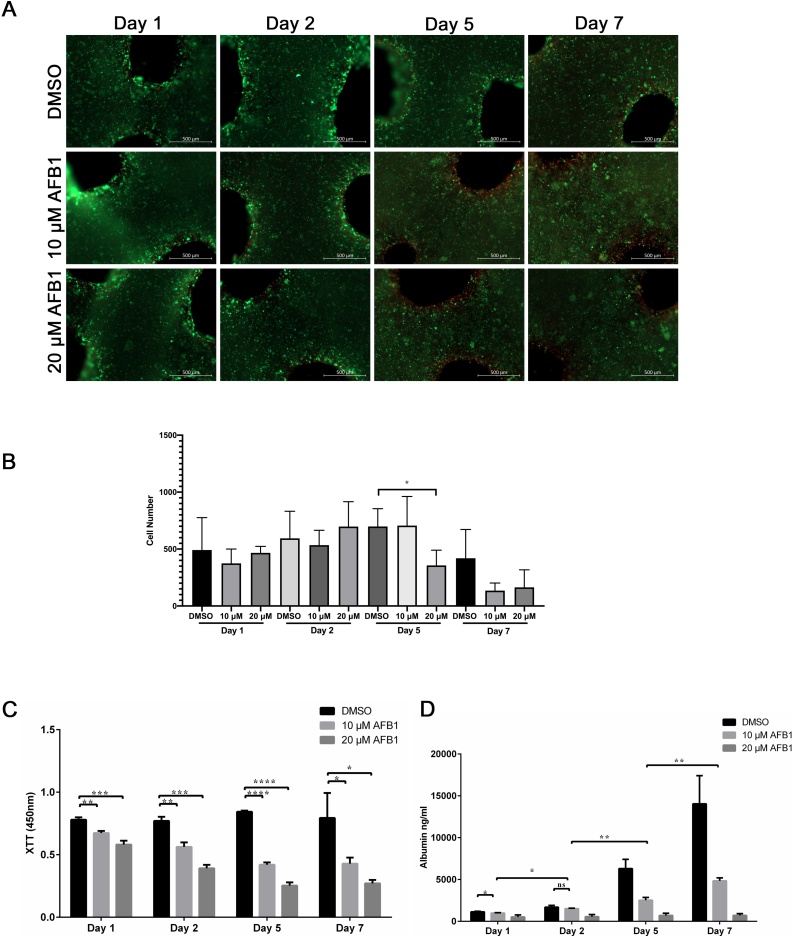
Effect of aflatoxin B1 on liver cells in 3D printed constructs. (**A**) Cell viability of mature HepaRG in 3D constructs upon treatment with AFB1 (10 μM or 20 μM) for the indicated time periods was visualized using the live-dead staining; calcein-AM (living cells in green) and ethidium homodimer-1 (dead cells in red). (**B**) Number of green-stained cells quantified with ImageJ in constructs treated with AFB1 for the indicated time points. Bars indicate the means ± SD, n ≥ 3 images. (**C**) Metabolic activity of the mature HepaRG cells treated with DMSO or AFB1 (10 μM or 20 μM) inside the 3D printed alginate/gelatin/Matrigel constructs was determined by the XTT assay at the indicated time points. Absorbance was measured at 450 nm. (**D**) Levels of albumin secreted from mature HepaRG cells in 3D constructs upon DMSO or AFB1 (10 μM or 20 μM) treatment were quantified at the indicated time points using ELISA analysis. Results are shown as mean ± SD of three independent experiments. * p ≤ 0.05; ** p ≤ 0.01; *** p ≤ 0.001; **** p ≤ 0.0001.

In 3D printed HepaRG constructs, a slight increase in sensitivity to AFB1 toxicity was apparent during acute and chronic exposure to the toxin using Live/Dead stain (Fig. 3A). Unlike the 2D cell culture model, AFB1 was not strongly toxic to the cells on days one and two (Fig. 3A). Further, AFB1 treatment did not show the substantial increases in toxicity over time that leads to the complete loss or death of the printed cells, which is evident from the strong green signal from the living cells and only small fraction of red, dead cells after five and seven days (Fig. 3A). Quantitative analysis of the number of green-stained cells showed that AFB1 treatment did not significantly decrease the number of living cells in all conditions except for constructs treated with 20 μM-AFB1 for 5 days (Fig. 3B). Our data suggest that printed HepaRG cells maintained in the 3D constructs and exposed to AFB1 had significantly higher viability after seven days, confirming their resistance against the hepatic cytotoxicity compared to 2D cell cultures.

We then sought to assess the metabolic activity of untreated and AFB1-treated cells in these models by quantifying the reduction of the tetrazolium salt XTT by dehydrogenase enzymes in the 3D constructs (Fig. 3C). For DMSO-treated 3D constructs, the XTT level increased to a minor extent over the one-week culture period. Further, AFB1 treatment slightly, but significantly, decreased the metabolic activity of the 3D constructs one day post-treatment (Fig. 3C). The level of metabolic activity two days post-treatment was similar to the metabolic level on day one post-treatment (Fig. 3C). Similarly, longer treatment (five- and seven-days post-treatment) showed an insignificant decrease in the metabolic activity of the 3D constructs in comparison to days one and day two.

To assess whether the metabolic capacity of the 3D printed HepaRG constructs was maintained upon AFB1 treatment, we quantified the albumin secretion to represent metabolic activity in untreated and treated 3D constructs using ELISAs. When comparing untreated and treated constructs, it was noticeable that AFB1 significantly inhibited the secretion of albumin at the indicated time points (Fig. 3D). However, a slight but significant increase of albumin secretion was observed in cells treated with 10 μM AFB1 at days five and seven in comparison to days one and two (Fig. 3D). In fact, when comparing the ratios of albumin concentration and metabolic activity in 2D and 3D microenvironments, the 3D constructs were more resistant than their 2D counterparts. The metabolic activity was found to be reduced thirteen-fold when comparing the control DMSO-treated cells with 20 μM AFB1 in 2D cultures after seven days. This reduction was only five-fold in the 3D constructs. Similar tendencies were found in albumin secretion; sixteen-fold reduction in 2D cultures, and only eight-fold reduction in 3D constructs when comparing DMSO and 20 μM AFB1 conditions. These results suggest an important role for AFB1 in the induction of cytotoxic effect in 3D bioprinted HepaRG cells, which perhaps explains the reduction of metabolic activity and albumin production of the liver cells upon treatment with the toxin.

**Fig. 4:**
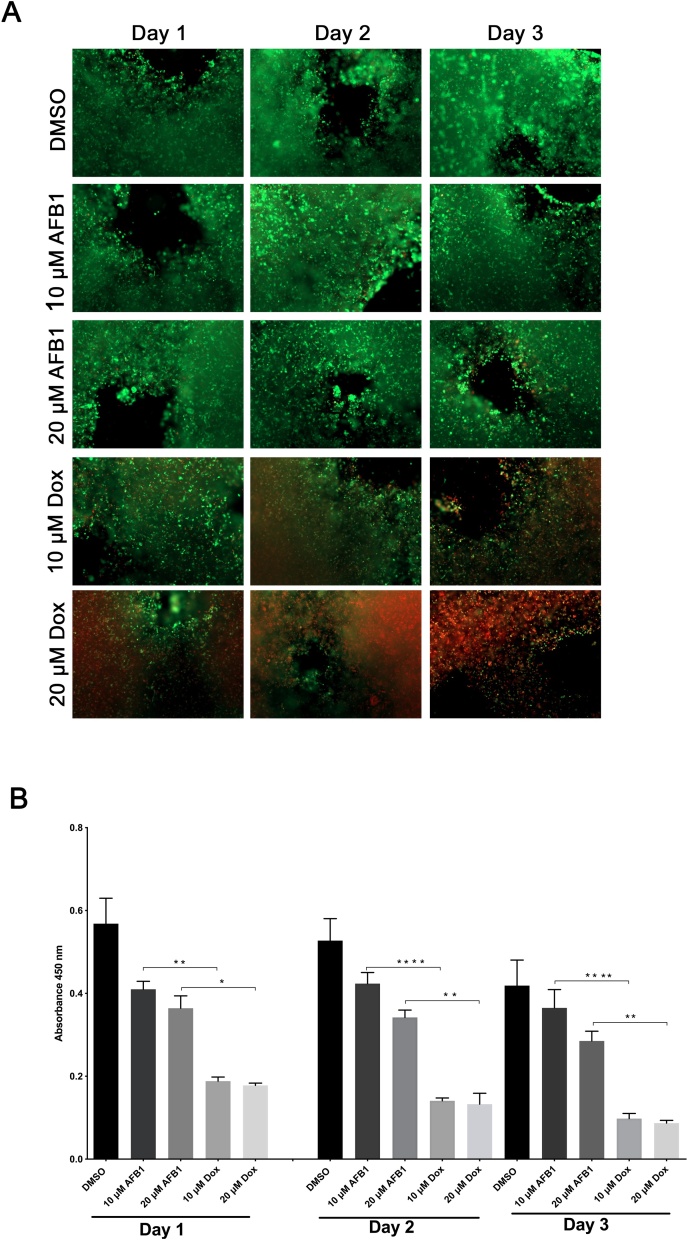
Effect of doxorubicin on liver cells in 3D printed constructs. (**A**) Cell viability of mature HepaRG in 3D constructs upon treatment with AFB1 or Dox (10 μM or 20 μM) for the indicated time periods was visualized using the live-dead staining; calcein-AM (living cells in green) and ethidium homodimer-1 (dead cells in red). (**B**) Metabolic activity of the mature HepaRG cells treated with DMSO, AFB1, or Dox inside the 3D printed constructs was determined by the XTT assay at the indicated time points. Absorbance was measured at 450 nm. Results are shown as mean ± SD of three independent experiments. * p ≤ 0.05; ** p ≤ 0.01; *** p ≤ 0.001; **** p ≤ 0.0001.

Next, we investigated whether prolonged survival of the 3D bioprinted HepaRG constructs was connected to the properties of the bioink used in the present study. We used doxorubicin (Dox) to check for cytotoxicity in HepaRG constructs. Indeed, Dox treatment induced strong cell death in the printed HepaRG cells three days post-treatment as visualized by microscopy using the live/dead stain (Fig. 4A), unlike AFB1 treatment. Additionally, metabolic activities of the printed HepaRG constructs were significantly reduced compared to AFB1 treated constructs (Fig. 4B). Thus, our data suggest that the bioink did not play a critical role in the cytotoxic effect of the toxins used in this study.

**Fig. 5:**
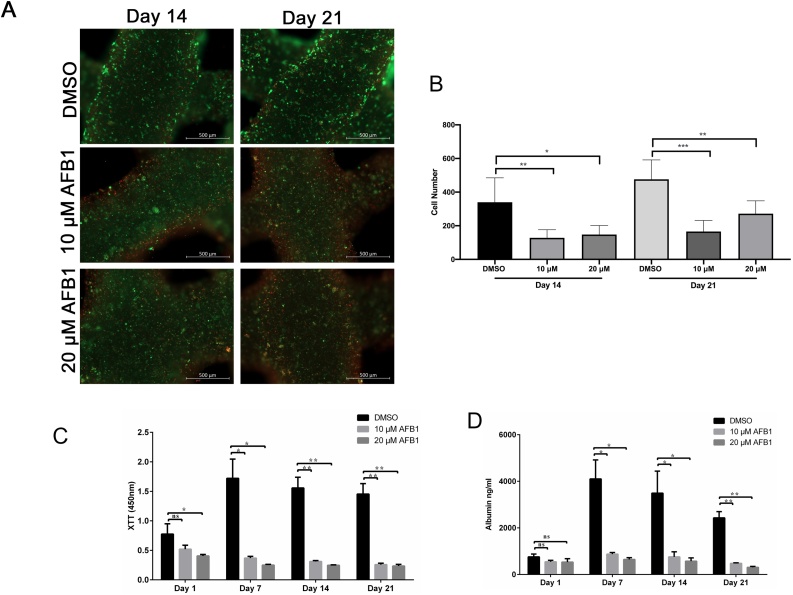
Long-term culture of mature HepaRG cells in 3D printed following aflatoxin B1 treatment. (**A**) 3D constructs treated with AFB1 (10 μM or 20 μM) for one week then constructs were cultured for additional 2 weeks with the toxin showed reduction in cell viability as visualized using the live-dead staining; calcein-AM (living cells in green) and ethidium homodimer-1 (dead cells in red). (**B**) Number of green-stained cells quantified with ImageJ revealed a significant decrease in number of living cells after two and three weeks of culture. Bars indicate the means ± SD, n ≥ 3 images. (**C**) Metabolic activity of the mature HepaRG cells treated with DMSO or AFB1 (10 μM or 20 μM) inside the 3D printed alginate/gelatin/Matrigel constructs was determined by the XTT assay at the indicated time points. Absorbance was measured at 450 nm. (**D**) Albumin secretion from mature HepaRG cells cultured in the 3D constructs upon treatment with DMSO or AFB1 (10 μM or 20 μM) was quantified at the indicated time points using ELISA analysis. Results are shown as mean ± SD of three independent experiments. * p ≤ 0.05; ** p ≤ 0.01; *** p ≤ 0.001; **** p ≤ 0.0001.

As 3D HepaRG constructs showed a more robust hepatic phenotype after one-week of AFB1 exposure, unlike the 2D culture, we sought to determine whether 3D printed HepaRG cells would survive a longer exposure to AFB1 (Fig. 5). Interestingly, cells in the printed 3D constructs were still viable two- and three-weeks after the initial treatment, as visualized by fluorescence microscopy using the Live/Dead stain (Fig. 5A). However, quantification of cells fluoresces in green revealed a significant decrease in number of living cells after two and three weeks of culture *in vitro* (Fig. 5B). In agreement with the Live/Dead stain data, the metabolic activity of cells in 3D constructs exhibited a slight reduction two- and three-weeks post-treatment, measured using XTT assays (Fig. 5C). Albumin levels two weeks post-treatment were similar to those measured one week post-treatment suggesting that cells are still viable and metabolically active (Fig. 5D). Taken together, functional characterization revealed that 3D-bioprinted HepaRG constructs exhibited a more robust hepatic phenotype than 2D cultures during prolonged AFB1 treatment and survived the cytotoxicity exerted by AFB1. This feature will enable longer-term and repeated exposure to allow the study of the hepatotoxicity of toxins, chemicals, and carcinogens.

## 4 Discussion

*In vitro* hepatic cell culture models based on 3D microenvironment have been shown to better reflect the *in vivo* behavior of liver cells than 2D cultures [27]. Models using 3D cell cultures have been used in clinical and basic research because they have proper polarization, cell-cell and cell-matrix contacts, and hepatic functions [28,29]. These advanced systems are currently being evaluated for infectious disease, toxicity testing, and cancer research [4,5]. Despite its great potential, bioprinting is still very challenging to use for toxicity testing in a 3D microenvironment post-printing. Thus, in this study we employed 3D bioprinting technology to study the feasibility and reproducibility of using 3D HepaRG constructs for toxicity testing *in vitro*. Bioprinted constructs were generated using the INKREDIBLE+3D-printer. The generated 3D constructs were highly viable, exhibited prolonged survival, reduced cell death, and prolonged secretion of albumin over the time course of culture *in vitro* compared to 2D cell cultures. Upon AFB1 treatment, 2D constructs reduced metabolic activity and increased cell death, which indicates a loss of hepatocyte functionality. However, 3D constructs were still viable and metabolically active but AFB1 treatment showed substantial increase in toxicity over time indicating that partial loss of hepatocytic activity. In contrast, AFB1 treatment resulted in complete destruction of the 2D monolayer within five days post-treatment. These 3D bioprinted liver constructs may thus serve for prolonged toxicity testing *in vitro*.

Several models including the traditional 2D cultures, 3D organoids, and spheroids have been employed using primary human hepatocytes, and the HepG2 and HepaRG cell lines for cytotoxicity testing *in vitro* [16,29,30]. It has been shown that 3D models including organoids and spheroids exhibit increased viability and functional stability compared to 2D monolayers, highlighting their utility for chronic toxicity studies *in vitro* [31]. Human primary hepatocytes are considered the gold standard for testing toxicity *in vitro* [32]. Yet primary spheroids or 3D models have been not considered for toxicity testing, in part, due to the difficulties in handling. In the present study, we used human HepaRG hepatocytes due to their metabolic competence to evaluate the appropriateness of 3D constructs for toxicity testing. Our 3D constructs exhibited long-term stability and cell viability, which is suitable for chronic toxicity testing *in vitro* similar to the 3D organoids and spheroids [33]. It has been shown that AFB1 treatment induces cell toxicity and loss of metabolic activity of HepaRG spheroid model ([34], [35]). Further, it has been reported that HepaRG spheroids were more sensitive to AFB1 exposure than the 2D culture [45]. This increased in toxicity was suggested to be correlated with different gene expression profile for cells cultured in 3D microenvironment. In agreement with our results, it has been also demonstrated that the metabolic activity of HepaRG 2D cell culture is highly reduced upon treatment with AFB1 *in vitro* [36]. Not only, AFB1 treatment diminished the metabolic activity of liver cells but also reduced albumin production in our 3D bioprinted cells as well as signs of stress and enhanced toxicity due to toxin treatment. However, no previous reports have investigated the direct of effect of AFB1 on albumin production *in vitro* using HepaRG cells and this needs further investigation. In conclusion, 3D models of liver cells result in an overall improvement of cell viability and are functionality suitable for chronic toxicity testing.

Animal models have been evaluated to acute and chronic doses of AFB1 toxicity *in vivo* [37,38]. Although, mice display low sensitivity to liver toxicity of AFB1 [39], a recent study has shown that a single dose of AFB1 can induce hepatic damage and inflammation in mice model. Moreover, the hepatic lipid droplets have been suggested to play crucial roles in the trapping, and detoxifying of AFB1 [37]. In contrast to mice models, rats are among the animals that are sensitive to AFB1that can induce liver damage [40]. Recently published data demonstrated that AFB1 induced hepatic damage through the alteration of gene expression of lipids in rats [38]. Interspecies differences in the susceptibility to AFB1-induced liver toxicity restrict the use of animal models for human related studies, therefore, 2D and 3D hepatic models are considered more reliable models for studies of AFB1 toxicity *in vitro*.

We used alginate/gelatin/Matrigel based-bioink which produced a stable matrix with a high porosity that sustained cell viability and metabolic activity of bioprinted cells, as previously described [26]. Blending the three components increased the printability, viability, and retained the shapes of the bioprinted constructs in the cultured medium post-printing. In our previous study, we demonstrated that the alginate/gelatin/Matrigel bioink has increased porosity using scanning electron microscopy. Thus, we chose to use the same blend composed of 2 % alginate, 3 % gelatin, and 20 % Matrigel for our bioink and printed the same cell number in a grid-shape constructs. Remarkably, the observed longer viability of 3D HepaRG cultures upon AFB1 treatment was not linked to the bioink composition, as doxorubicin induced strong toxicity against the 3D cultures, suggesting the suitability of this bioink in testing for toxicity *in vitro*.

In conclusion, our data presented here indicate that 3D bioprinting using HepaRG cells extends cellular function *in vitro*, relative to 2D cell cultures. Our data corroborates previous findings, which demonstrated that liver cells can survive for longer periods in a 3D microenvironment post-printing [20], in which cytotoxicity in the 3D constructs was continuously monitored over a long period of time; up to 3 weeks. Although it is still not clear how HepaRG cells in 3D bioprinted constructs can sustain cell viability upon AFB1 treatment for so long, we believe that our work highlights the crucial role of cell-cell and cell-matrix contact for epithelial cell function and viability, which sustain the epithelial phenotype and provide a 3D microenvironment that enhances cell resistance to toxicity exerted by toxins *in vitro* such as AFB1 [41]. Therefore, for cytotoxicity testing, responses may be more adequately simulated in liver 3D bioprinted models.

## Author statement

K.S. performed the experiments. V.R. helped with the conduction of the albumin ELISA. J.B. contributed to the analysis of the data. M.A.A.Z. conceived the project, performed the experiments, analyzed the data, and wrote the manuscript. J.K. supervised the project and wrote and edited the manuscript.

## Declaration of Competing Interest

The authors report no declarations of interest.
